# ONS: an ontology for a standardized description of interventions and observational studies in nutrition

**DOI:** 10.1186/s12263-018-0601-y

**Published:** 2018-04-30

**Authors:** Francesco Vitali, Rosario Lombardo, Damariz Rivero, Fulvio Mattivi, Pietro Franceschi, Alessandra Bordoni, Alessia Trimigno, Francesco Capozzi, Giovanni Felici, Francesco Taglino, Franco Miglietta, Nathalie De Cock, Carl Lachat, Bernard De Baets, Guy De Tré, Mariona Pinart, Katharina Nimptsch, Tobias Pischon, Jildau Bouwman, Duccio Cavalieri, the ENPADASI consortium

**Affiliations:** 1 Institute of Biometeorology (IBIMET), National Research Council (CNR), Via Giovanni Caproni, 8, 50145 Florence, FI Italy; 2 The Microsoft Research - University of Trento Centre for Computational and Systems Biology (COSBI), Piazza Manifattura, 1, I-38068 Rovereto, TN Italy; 3 Department of Food Technology, Safety and Health, Ghent University, Coupure links 653, 9000 Ghent, Belgium; 4 Department of Biology, University of Florence, Via Madonna del Piano, 6, 50019 Sesto F, FI Italy; 5 Food Quality and Nutrition Department, Research and Innovation Centre, Edmund Mach Foundation, Via Edmund Mach, 1, 38010 San Michele all’Adige, TN Italy; 6 Department of Agri-Food Sciences and Technologies, University of Bologna, Piazza Goidanich 60, Cesena, FC Italy; 7 Institute for Systems Analysis and Computer Science (IASI), National Research Council (CNR), Via dei Taurini, 19, 00185 Rome, RM Italy; 8 KERMIT, Department of Data Analysis and Mathematical Modelling, Ghent University, Coupure links 653, 9000 Ghent, Belgium; 9 Department of Telecommunications and Information Processing, Ghent University, Coupure links 653, 9000 Ghent, Belgium; 10 Molecular Epidemiology Research Group, Max Delbrück Center for Molecular Medicine, Berlin, Germany; 11 Microbiology and Systems Biology, TNO, Utrechtseweg 48, 3704HE Zeist, The Netherlands; 12 Center Agriculture Food Environment, University of Trento, San Michele all’Adige, Italy

**Keywords:** Ontology, Nutrition, Health, Intervention study, Observational study, Metabolomics, Food intake, Biomarker, Databases

## Abstract

**Background:**

The multidisciplinary nature of nutrition research is one of its main strengths. At the same time, however, it presents a major obstacle to integrate data analysis, especially for the terminological and semantic interpretations that specific research fields or communities are used to. To date, a proper ontology to structure and formalize the concepts used for the description of nutritional studies is still lacking.

**Results:**

We have developed the Ontology for Nutritional Studies (ONS) by harmonizing selected pre-existing de facto ontologies with novel health and nutritional terminology classifications. The ONS is the result of a scholarly consensus of 51 research centers in nine European countries. The ontology classes and relations are commonly encountered while conducting, storing, harmonizing, integrating, describing, and searching nutritional studies. The ONS facilitates the description and specification of complex nutritional studies as demonstrated with two application scenarios.

**Conclusions:**

The ONS is the first systematic effort to provide a solid and extensible formal ontology framework for nutritional studies. Integration of new information can be easily achieved by the addition of extra modules (i.e., nutrigenomics, metabolomics, nutrikinetics, and quality appraisal). The ONS provides a unified and standardized terminology for nutritional studies as a resource for nutrition researchers who might not necessarily be familiar with ontologies and standardization concepts.

**Electronic supplementary material:**

The online version of this article (10.1186/s12263-018-0601-y) contains supplementary material, which is available to authorized users.

## Background

Human Nutritional Science studies the effects of food components on metabolism, health, performance, and disease resistance of humans, also encompassing the study of human behavior related to food choices. Nutritional epidemiology, on the other hand, assesses the relations between diet, nutrients and health, and disease outcomes [1]. Yet, there is a major disconnection between the description of nutrition-based prevention of disease and the understanding of the complex network of interactions by which nutrition modulates health. To fill this gap, a set of nutrition-related sub-disciplines (e.g., nutritional biochemistry, clinical nutrition, nutritional epidemiology, nutrigenetics, and nutrimetabolomics) provide fundamental evidence at different levels and from different perspectives, contributing to the expansion of nutritional science as a more systematic and complex discipline [2, 3]. As nutrition data are heterogeneous in terms of quality and nature, a comprehensive consideration of all aspects is challenging [4], even if substantial advance has been made to improve the reporting of findings and the data quality [5] of nutrition research [6], which is one of the prerequisites for integrated analysis.

To integrate evidence, a systematic re-organization of concept definitions is needed. Currently, concept definitions are often derived from multiple sources, with the drawback that slight variations can lead to misleading interpretations [7]. Since in bioscience in general, and in nutritional science in particular, the same concept can be referred to by multiple synonymous terms, abbreviations, or acronyms [8], as well as using different languages, term classifications such as the Medical Subject Headings (MeSH) [9] or the NCI Thesaurus [10] provide fundamental resources. However, thesauri or controlled vocabularies for biomedical information do not specify relations between concepts. Although those efforts can be used to standardize general study descriptions, considerable advances would arise from the use of resources that, in addition to standardizing the vocabulary, also include connections/relations between classes, such as ontologies, specifically tailored to the nutritional sciences.

Often biomedical researchers refer to ontologies using the terminologies more appropriately pertaining to “controlled vocabularies,” “thesauri” (i.e., a list, often organized in a hierarchy or taxonomy, of concepts and their textual descriptions), or “taxonomies” (i.e., a hierarchy consisting of terms denoting classes linked by sub- and super-class relations). A proper ontology, however, is defined as a formal representation of knowledge in a certain reality (i.e., a certain domain of knowledge), in a way that different people—and, notably, computers—can understand the concepts it contains and learn about the reality that is being represented [8, 11]. Ontologies consist of defined classes of entities, typically structured within a knowledge hierarchy where concepts are connected by standardized [12] semantic relationships (i.e., “is-a,” “part-of”) formally specifying knowledge relations such as generalizations of specifications of the reality of interest [13].

Open Biomedical Ontologies (OBO), established in 2001, is a platform for developing interoperable ontologies for biomedical research [14]. Efforts have been made in the agricultural field to develop nutrition-oriented ontologies focused on the description of food components such as “the food classification and description system” [15] developed by European Food Safety Authority (EFSA). Other notable efforts in developing food-focused ontologies were reviewed elsewhere [16]. Based on literature search and public ontological repository queries (OBO Foundry searched using ONTOBEE, and Bioportal), a single example of a nutritional ontology was found (the Bionutrition Ontology—BNO, http://purl.bioontology.org/ontology/BNO). The latter represents a controlled vocabulary of nutritional terms, without a proper annotation of terms or definition of properties, and lacks orthogonality (i.e., no terms are imported or refer to external ontologies). To the authors’ knowledge, a proper ontology integrating the terms related to food description, medical science, genetics, genomics data, and nutritional science methods for diet and health research is not available to date. To fill this gap, we present the Ontology for Nutritional Studies (ONS) to facilitate the harmonization and integration of biological samples collected using different methodologies, referred to by differing terminologies in various fast-growing sub-disciplines in the dietary and health research.

The ONS was developed within the European Nutritional Phenotype Assessment and Data Sharing Initiative (ENPADASI) consortium [17], which joins scientists from 51 research centers in nine countries of Europe with the common effort to handle and make available big nutritional data through the open access nutritional database Data Sharing In Nutrition (DASH-IN) [17, 18]. DASH-IN is a distributed pan-European infrastructure and supports the storage of both interventional and observational studies and provides the tools for distributed management and search and analysis of the data [19]. The development of this infrastructure requires an ontology to harmonize biochemical, genetic, clinical, and nutritional concepts typically found in intervention and observational studies. The ontology would provide a coherent means of data annotation and data querying over the distributed infrastructure. Further developments of the project led to a stronger need for a proper conceptual framework such as the ONS that could be used by a broader nutrition community to build upon for annotating general nutritional studies. The ENPADASI framework gathered researchers from different nutrition-related fields (health sciences, biology, genetics, microbiology, agricultural sciences, food technology, science of materials, chemistry, metabolomics, genomics, bioinformatics, and metagenomics) and offered the ideal milieu for creating the first ontology in nutrition.

## Methods

Terms to be included in the ONS were collected among partners of the ENPADASI consortium, as well as from templates for data and metadata upload into the DASH-IN databases. In compliance with the OBO Foundry principles [14], the ONS has been developed to be as follows: (i) Interoperable with other ontologies, as it has been formalized using the latest OWL 2 Web Ontology Language [20] and RDF specifications [21] and edited using Protégé [22]; the hermit reasoner (http://hermit-reasoner.com/) was used for consistency checking. (ii) Accessible, under the Creative Commons license (CC BY 4.0), published on GitHub (https://github.com/enpadasi/Ontology-for-Nutritional-Studies) and at NCBO BioPortal (http://bioportal.bioontology.org/ontologies/ONS). (iii) Orthogonal to other ontologies by reusing existing terms. Besides assuring compliance with the OBO Foundry principles, we also ensured that the ONS followed the increasingly established FAIR principles [23]. As such, the ONS is also published in the FAIRsharing database (https://fairsharing.org/bsg-s001068).

To enhance interoperability with other ontologies, the ONS builds on a subset of the Ontology for Biomedical Investigations (OBI) [24]. The subset was created using the ONTODOG tool [25] and is composed of all terms relevant to nutritional investigations and extended also in accordance with the bioinformatics infrastructure of ENPADASI. Moreover, this assured the adoption of a well-defined and widely adopted structure for the top and mid-level classes and principally the adherence to the Basic Formal Ontology (BFO) [26] as upper ontology.

Additional relevant ontologies were used orthogonally in the ONS as discussed in the results. To ensure and enhance orthogonality, all terms were first searched using the ONTOBEE [27] web service and catalogued with their URIs. ONTOFOX [28] was then used to import all terms with related annotations and axioms (option includeAllAnnotations). Newly defined terms, specific to the ONS, have been labeled with “ONS_” followed by a 7-digit number. Terms related to food description were also included by importing a subset of terms from the FOODON ontology [29]. All intermediate files of this development process (i.e., template files used for web services or imported ontologies) were stored on GitHub repository. Additional file 1 contains instruction on how to browse, download, and contribute to ONS. The same instruction is also present online at the wiki page of the GitHub repository (https://github.com/enpadasi/Ontology-for-Nutritional-Studies/wiki). In this development process, terms from a number of different ontologies were imported. Table 1 reports a summary of the classes that were imported in the ONS (excluding individuals) and their ontology of origin.

**Table 1:** Prefix and URL of the ontology of origin of the classes imported in ONS

	Prefix	No. of terms in ONS	URL
Basic Formal Ontology	BFO	45	http://ifomis.uni-saarland.de/bfo/
Chemical Entities of Biological Interest	CHEBI	14	https://www.ebi.ac.uk/chebi/
Clinical Measurement Ontology	CMO	5	https://bioportal.bioontology.org/ontologies/CMO
EMBRACE Data And Methods Ontology	EDAM	12	http://edamontology.org/page
Experimental Factor Ontology	EFO	30	https://www.ebi.ac.uk/efo/
eagle-i Research Resource Ontology	ERO	2	https://open.med.harvard.edu/wiki/display/eaglei/Ontology
Food Ontology	FOODON	2809	https://foodontology.github.io/foodon/
Human Phenotype Ontology	HP	2	http://human-phenotype-ontology.github.io/
The Information Artifact Ontology	IAO	63	https://github.com/information-artifact-ontology/IAO
Informed Consent Ontology	ICO	5	https://github.com/ICO-ontology/ICO
NCBITaxon ontology	NCBITaxon	17	http://www.obofoundry.org/ontology/ncbitaxon.html
The NCI Thesaurus	NCIT	26	http://www.obofoundry.org/ontology/ncit.html
Ontology of Biological and Clinical Statistics	OBCS	5	https://github.com/obcs/obcs
Ontology for Biomedical Investigations	OBI	265	http://obi-ontology.org/
Ontology for Biobanking	OBIB	4	http://www.obofoundry.org/ontology/obib.html
Ontology for General Medical Science	OGMS	11	http://www.obofoundry.org/ontology/ogms.html
The Ontology of Host-Microbiome Interactions	OHMI	4	https://github.com/OHMI-ontology/OHMI
Ontological Minimum Information About BIobank data Sharing	OMIABIS	3	http://www.obofoundry.org/ontology/omiabis.html
The Ontology of Medically Related Social Entities	OMRSE	1	https://github.com/ufbmi/OMRSE
The Semanticscience Integrated Ontology	SIO	33	https://github.com/micheldumontier/semanticscience
STATistics Ontology	STATO	4	http://stato-ontology.org/
An ontology of units of measurements	UO	18	https://github.com/bio-ontology-research-group/unit-ontology

## Results

The initial ontological curation identified a large number of relevant terms to consider. The terms were then either imported from existing ontologies, redefined from existing concepts, or annotated de novo. By merging 3334 terms imported from already existing ontologies and 100 newly defined terms, the ONS describes both intervention and observational studies in nutrition.

### Central nutritional concepts

**Fig. 1:**
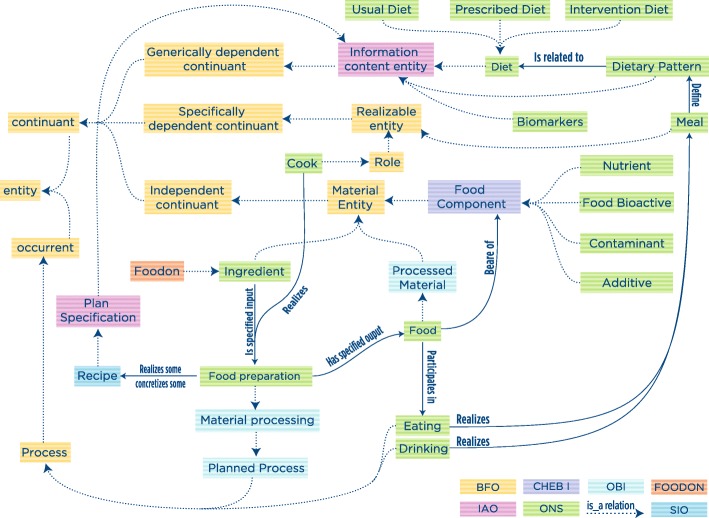
Upper and mid-level hierarchical structure of the ONS showing how relevant nutritional concepts have been related to each other. Considering the large number of concepts and relations defined in the ONS, further detailed relations are not shown here. The terms in green boxes are ONS-specific terms, while terms in other color boxes are imported from existing ontologies (i.e., BFO, OBI, IAO, CHEBI, SIO, FOODON). Dotted arrows represent “is_a” semantic relations, while solid arrows represent other types of semantic relations

In the ONS, relevant nutritional concepts have been related to each other to offer a well-organized synopsis of the knowledge in health and nutrition sciences. The ONS harmonizes all pertinent concepts from different domains, defining appropriate relationships and improving and simplifying the process of conceptual organization of the many facets of real studies. Here, we present (Fig. 1) how diet, food, and food component concepts, which can be considered central for an ontology aimed at effectively assisting researchers in the standardized description of the nutritional study they are conducting, were included, defined, and connected in the ONS.

Diet is defined as the regular course of eating and drinking adopted by a person or animal (ONS_0000080). For the purpose of the nutritional community, we further detailed the diet concept into three sub-classes: (i) Usual diet is defined as the regular course of eating and drinking adopted by a population in a certain geographical area, or in a certain cultural setting, or following certain common eating behavior. It is also intended as the diet a person would follow without further prescription or indications, i.e., vegetarian diet (ONS_0000083). (ii) Prescribed diet is defined as a diet prescribed by a physician/nutritionist to meet specific nutritional needs of a person (ONS_0000082). (iii) Intervention diet is defined as the diet administered during an intervention study. It usually comprises the adoption of a certain nutritional intervention (ERO_0000347), intended as the prescription of consuming or not consuming certain food, and follows a precise study design. Intervention studies usually compare at least two subgroups of a population, one control group receiving a null nutritional intervention and one or more test groups receiving the intervention (ONS_0000081).

Food component is defined as any substance that is distributed in foodstuffs. It includes materials derived from plants or animals, such as vitamins or minerals, as well as environmental contaminants (CHEBI_78295, ONS_0000073). Starting from this definition, we further detailed the food component concept into different sub-classes: (i) Nutrient (ONS_0000077): A nutrient is a food component used by the body for normal physiological functions that guarantee survival and growth. It must be supplied in adequate and defined amounts from foods consumed within a diet. Malnutrition occurs when the right amount of nutrient is not provided. (ii) Food bioactive (ONS_0000076): A food bioactive is a food component other than those needed to meet basic human nutritional needs (nutrients). Food bioactives modulate one or more metabolic processes, possibly resulting in the promotion of better health. The daily required intake for food bioactives is not established yet, and there is no demonstration that malnutrition occurs when the right amount is not provided. (iii) Contaminant: Contaminant is unwanted food component that makes the food no longer suitable for use (ONS_0000075). (iv) Additive: Additive is a component added to food to improve or preserve it (ONS_0000074).

Multiple definitions can be found for the food concept. As an example, CHEBI (CHEBI:33290) defines “Any material that can be ingested by an organism” and MESH (MeSH D005502) defines “Any substances taken in by the body that provides nourishment.” For the purposes of the nutritional community, the concept of food was expanded as food is defined as a complex matrix that is consumed by a person through the process of eating or drinking (ONS_0000079). Foods are bearer of the nutrients, bioactives, and, sometimes, other food components. Food consumption, through the meal consumption, follows a certain dietary pattern, which define the diet. Nutrients and bioactives contained in food can be exploited by the human organism thanks to the process of digestion (ONS_0000101), absorption (ONS_0000102), metabolization (ONS_0000103), or through the intervention of the gut microflora (OHMI_0000020). The concept of food can be split into the following: (i) Raw food: A raw food is an uncooked, unprocessed food that is consumed in its natural state (ONS_0000099); (ii) Processed food: A processed food is the result of the process of home or industrial food preparation (ONS_0000100).

In nutritional science, biomarkers are increasingly being used to provide objective results and to avoid biases (e.g., reporting bias and recall bias). Three groups of biomarkers were identified for use in nutrition science [30], along with the dietary biomarker development framework: “exposure biomarker” for dietary intake and nutrient status, “effect biomarker” for measuring biological effects of food components, and “susceptibility biomarker” for assessing the effects of diet on human health. In the ONS, we are presenting the first formal ontology application for the biomarker class (ONS_0000095) and its sub-classes, using the definition from the commentary [30]. ONTOBEE query for the “biomarker” returned multiple results mainly from the Experimental Factor Ontology (EFO), all having the class “Measurement” (EFO_0001444) as super-class (a measurement is an information entity that is a recording of the output of a measurement such as produced by an instrument). However, it has to be noted that a similar class can also be found in the Information Artifact Ontology (IAO) named “Measurement datum” (IAO_0000109, a measurement datum is an information content entity that is a recording of the output of a measurement such as produced by a device). In the ONS, the biomarker class was defined as a sub-class of the “Measurement datum” class (IAO_0000109) in line with the OBI ontology, which uses the IAO class.

Integrated analysis of data and joint pooled analysis are strongly promoted in nutrition by research funders, though raise scientists’ concern, as the scientific interest in the open access to nutritional data often conflicts with the General Data Protection Regulation. When fully achieved, integrated analysis will lead to new discoveries and maximize use of public funds. In ENPADASI, this problem was broadly dealt with from both legal and technical aspects, and a recommendation on minimal information to be added as metadata to studies to boost integration capacity has been developed [19]. The identification of minimal requirements, essential to connect existing and future study (meta) databases, facilitates data exchange and data interpretation, helping to increase the robustness of results from future joint data analysis in nutritional epidemiology [31]. In fact, joint data analysis has already started helping to achieve new discoveries [32]. In the ONS, we have included the minimal required study information in the growing conceptual/ontological framework. Each minimal required study term was placed at the appropriate hierarchical level in the ontology. To easily identify terms pertaining to the minimal study information, an annotation property (“in_minimal_requirements_subset”) was created.

### Application scenarios

**Fig. 2:**
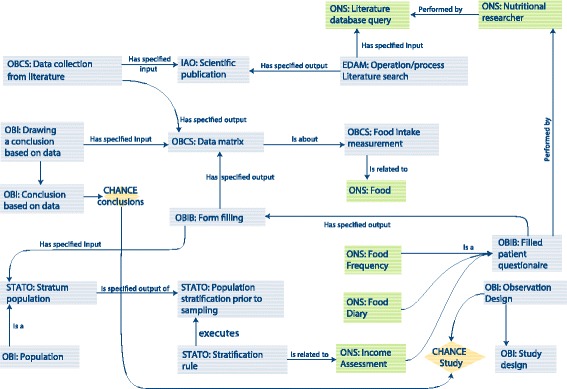
Application scenario to the description of an observational study: modeling of the CHANCE study with the ONS. Terms in rhombus indicate instance-level terms specific to the CHANCE study (i.e., the specific conclusion of the CHANCE study), while terms in rectangular boxes represent general concept in the ONS. The presented semantic representation should be intended at the single instance level for the purpose of specifically describe CHANCE study

**Fig. 3:**
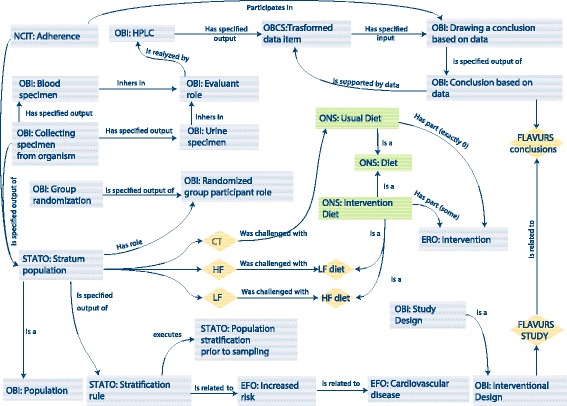
Application scenario to the description of an intervention study: modeling of the FLAVURS study with the ONS. Terms in rhombus indicate instance-level terms specific to the FLAVURS study (i.e., the specific conclusion of the FLAVURS study), while terms in rectangular boxes represent general concept in the ONS. The presented semantic representation should be intended at the single instance level for the purpose of specifically describe FLAVURS study

The ONS is designed to enable the description of both intervention and observational studies in human nutrition. Here, we present two application scenarios based on published nutritional studies, one for the observational study design and one for the interventional study design. Figures 2 and 3 illustrate how the ONS was built to support the standardized annotation of most descriptors of a nutritional study, starting from initial phases of a study (i.e., formalizing the definition of population stratum) to finally connect to the specific results and how they were obtained. Figures and descriptions have to be intended at the single instance level (i.e., specific for the study object of description). For this reason, we introduced the use of individuals (and their connections) for very study-specific element alongside concepts in classes. In the text below, the italic notation indicates the properties, while the notation PREFIX:CLASS is used to indicate classes in the ontology, for example the notation “ONS:Diet” indicates the class with label “Diet” in the ONS ontology. For abbreviation of the ontologies, we refer the reader to the list of imported ontologies in the “Methods” section.

### Observational studies

The first application scenario is represented by the CHANCE study [33]. Figure 2 illustrates how the ONS can be used to formalize information on how the study was conducted. This observational study aims at developing novel and affordable nutritious foods to optimize the diet and reduce the risk of diet-related diseases among groups at risk of poverty (ROP). The CHANCE study uses two different approaches to draw its final conclusion. The first is a literature search process (EDAM:Literature search), performed with a specific textual literature database query (i.e., an instance of the class ONS:Literature database query). Output of the literature search process is a number of scientific publications (IAO:Scientific publication) which are subject to analysis and review to extract data (OBCS:data collection from literature), a process that ultimately results in an organized data matrix (OBCS:Data matrix). CHANCE also included an observational study approach. In this case, a population was firstly divided into sub-populations based on their economic income. This stratification (STATO:Population stratification prior to sampling) was carried out following a specific stratification rule (STATO:Stratification rule), based on the risk of poverty (ROP) of the subjects assessed with a questionnaire (ONS:Income assessment). The stratified population was then challenged with (i.e., *is specified input of*) two nutritional questionnaires (ONS:Food frequency and ONS:Food diary) aimed at assessing the foods consumed by the subjects and producing results finally organized in a data matrix. In both cases, the data matrices (OBCS:Data matrix) specific for this study contain information about the nutrients and food consumed by the population and represent the specified data object on which conclusions are drawn (OBI:drawing a conclusion based on data).

### Intervention studies

The second application scenario is represented by the FLAVURS (impact of increasing doses of flavonoid-rich and flavonoid-poor fruit and vegetables on cardiovascular risk factors in an ‘at risk’ group) study [34]. Figure 3 illustrates how the ONS can be used to formalize the information on how the study was conducted. This interventional study aimed to investigate the effects of high and low flavonoid diets on the vascular function and other cardiovascular disease risk factors. In this study, a population, selected on the basis of the stratification rule (STATO:Stratification rule) of having a relative risk of developing cardiovascular disease higher than 1.5, has been randomly divided (OBI:Group randomization and OBI:Randomized group participant role) into three groups: control group (CT), high flavonoid group (HF), and low flavonoid group (LF). Each of the groups was challenged with a different diet (ONS:Diet): CT followed the usual diet (ONS:Usual Diet), which is defined to *have exactly 0* interventions (ERO:Intervention); in the HF and the LF groups, individuals were challenged with two different types of intervention diet (ONS:Intervention diet) encompassing two different intervention (ERO:Intervention) protocols. In HF diet, the intervention was performed by the prescription of consuming fruit and vegetables with high flavonoid content, while in the LF diet the intervention was concretized by the prescription of consuming fruit and vegetables with low flavonoid content.

Urine and blood (OBI:Urine specimen and OBI:Blood specimen) were collected from individuals (OBI:Collecting specimen from organism) and analyzed (i.e., they *inherited* the evaluant role OBI:Evaluant role) by an HPLC assay (HPLC class) including untargeted metabolomics [35]. Output of the analysis was a data item in the form of a matrix (OBCS:Transformed data item) that is used to draw specific FLAVURS conclusions (OBI:Drawing a conclusion based on data and OBI:conclusion based on data).

## Discussion and conclusions

The ONS is the first systematic effort to provide a formal ontology framework for the description of nutritional studies. In this context, the main aim of the ONS is the establishment of an ontological framework that can assist nutrition researchers by selecting the appropriate terms from the wide range of existing ontologies and creating the relevant missing key concepts for the field. Nutrition researchers, who might not necessarily be familiar with ontologies and concept standardization, can find in the ONS a single knowledge entry point for a unified and standardized terminology without having to resort to numerous ontology sources. In addition to standardizing concept descriptions and assisting in annotation, the ONS will structure querying of nutritional studies stored in public databases (such as the resources developed in the ENPADASI project). Finding the suitable studies (i.e., those more directly comparable regarding design, employed stratification criteria, or type of intervention diet employed) represents the basis for integrated analysis. Such a query, in fact, cannot be efficiently based on string matching, but rather on more complex textual analysis and machine learning methodologies for which ontology is crucial. A well-established nutritional ontology would also enable more accurate search for required data as well as the automated integration and analysis of data from multiple sources [36].

Diet, nutrient, and food are indeed central concepts for nutritional sciences, and they were included and connected with higher level concepts in ONS. Moreover, the ONS supports the research needs identified by other initiatives such as the Food Biomarkers Alliance (FoodBAll) by including for the first time in a formal ontology the concept of biomarker in nutrition, and its sub-classes, as defined in [30].

Besides acquiring widespread utilization, an ontology can be considered successful only if (i) continuous development and (ii) constant contribution/updates from researchers with specific knowledge is ensured. We invite and encourage researchers in the nutritional field to contribute to the further development, adoption, and promotion of the ONS. Contributions are already possible using the GitHub tracking/issues system (Additional file 1) and an online community platform to facilitate the process of curation and extension of the ONS will be developed for this purpose. As a next challenge, the ONS aims to integrate nutritional studies with non-life sciences such as economy, psychology, and sociology, which also influence the nutritional status of individuals [37–39].

## Additional file

Additional file 1: Agile introduction on ontologies, how to use them and contribute to ONS. (PDF 2119 kb)

## Abbreviations

DASH-IN: Data Sharing In Nutrition
EFSA: European Food Safety Authority
ENPADASI: European Nutritional Phenotype Assessment and Data Sharing Initiative
FLAVURS: Impact of increasing doses of flavonoid-rich and flavonoid-poor fruit and vegetables on cardiovascular risk factors in an “at risk” group
FoodEx2: Version 2 of the EFSA Food classification and description system for exposure assessment
HPLC: High-performance liquid chromatography
MeSH: Medical Subject Headings
OBO: Open Biomedical Ontologies
ONS: Ontology for Nutritional Studies
OWL: Web Ontology Language
RDF: Resource Description Framework
ROP: Risk of poverty
