# Supplementary material for: ONS: an ontology for a standardized description of interventions and observational studies in nutrition
Source: Genes Nutr. 2018 Apr 30;13:12. doi: 10.1186/s12263-018-0601-y (PMC5928560; doi:10.1186/s12263-018-0601-y)
Supplement: Supplementary file 1 — Agile introduction on ontologies, how to use them and contribute to ONS. (PDF 2119 kb) [file 12263_2018_601_MOESM1_ESM.pdf]

Supplementary material to manuscript GNNU-D-18-00006

TITLE

ONS: an ontology for a standardized description of interventions and observational studies in nutrition

AUTHORS

Francesco Vitali, Rosario Lombardo, Damariz Rivero, Fulvio Mattivi, Pietro Franceschi, Alessandra Bordoni, Alessia Trimigno, Francesco Capozzi, Giovanni Felici, Francesco Taglino, Franco Miglietta, Nathalie De Cock, Carl Lachat, Bernard De Baets, Guy De Tré, Mariona Pinart, Katharina Nimptsch, Tobias Pischon, Jildau Bouwman, Duccio Cavalieri and the ENPADASI consortium.

The aim of this document is to help users of the Ontology for Nutritional Studies (ONS) to use, browse, and contribute to the ontology. This guide is also available online at the GitHub wiki pages of ONS (https://github.com/enpadasi/Ontology-for-Nutritional-Studies/wiki). Most of the documentation is summarised and put together from external resources, whom shall go the credits.

## TABLE OF CONTENT

1. General introduction on ontologies
2. ONS: Browse and lookup ONS concepts
3. ONS: Browsing and making changes to ONS locally
4. ONS: contribute to ONS

## GENERAL INTRODUCTION ON ONTOLOGIES

An ontology is defined as a formal representation of the knowledge in a certain reality (i.e. a certain domain of knowledge), in a way that different people - and, notably, computers - can understand the concepts it contains and learn about the reality that is being represented. Ontologies consist of defined classes of entities, typically structured within a knowledge hierarchy where concepts are connected by standardised semantic relationships (i.e. "is-a", "part-of") formally specifying knowledge relations such as generalisations of specifications of the reality of interest.

In ontological jargon we can identify some main components and features. Here we will use a classical example in ontological field, an extract from this guide (https://protege.stanford.edu/publications/ontology_development/ontology101-noy-mcguinness.html), to illustrate the component of an ontology and to provide practical directions.

**Classes:** are the concepts in a domain of discourse. For example, if our domain of discourse is represented by wine and winery, the "wine" class would represent all possible wines. In an ontology, classes are organized in a hierarchical structure of subclasses and superclasses. **Subclasses** of a certain class represent concepts that are more specific than their **superclass**. For example, we can divide the class of all wines into red, white, and rosé wines (i.e. the main wine styles). The raw organization of classes and subclasses represents the "skeleton" of the ontology, indicating the basic relationships that connect the classes of the ontology. In a structure like this, classes at various depth in the hierarchy can be identified, form the broader top-level concepts (i.e. wine, a class which comprises all the wines) to the most granular ones at the bottom level (i.e. a Red Bordeaux is a sub-class of Red wine, which in turn is a subclass of Wine).

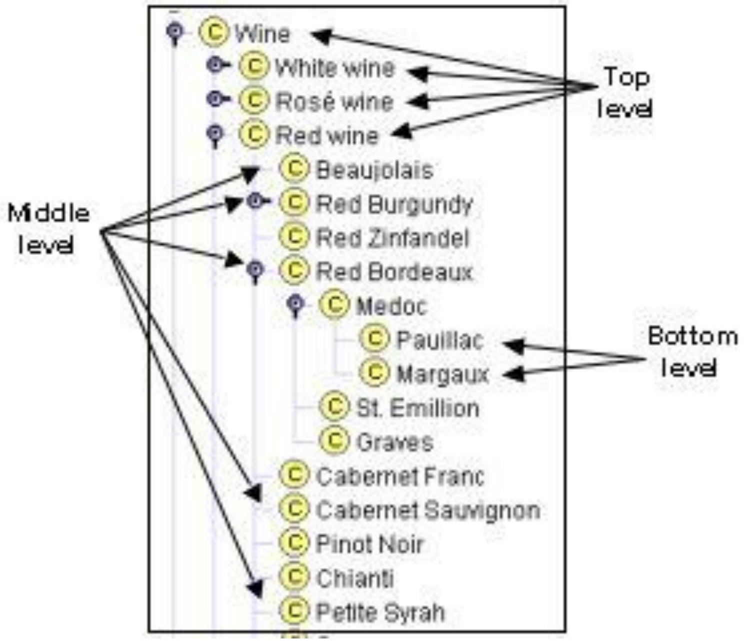


Figure 1: Classes of the wine ontology organized in a hierarchy. All of the terms are classes of the ontology, and the tree-like structure defines the defines the super and sub-class relationships

**Instances:** while classes of the ontology should have universal meaning, to define concepts universally applicable, instances of a class represent specific individuals of that class, which cannot have universal

meaning. In our wine example, the red Bordeaux produced by a certain winery, or the red Bordeaux in this glass of wine, are instances of the Red Bordeaux subclass.

**Properties:** classes, subclasses and their individuals are the concepts of the domain of discourse that the ontology is representing. **Properties** describe the various features and attributes of those concepts and how they relate to each other's. The basic type of properties, which has been already introduced in the example above, are those specifying the super and subclass relationships among classes. Formally, those properties would be read as "is_a" property connecting each subclass to its superclass

For the purposes of organising the concepts in biomedical and scientific domain ontologies, the Basic Formal Ontology (BFO) comes handy (http://ifomis.uni-saarland.de/bfo/). The BFO is a small, general and genuinely upper-level, ontology aiming at dividing the reality in defined classes, which could be applied to any domain of discourse. It does not contain physical, chemical, biological or other terms which would properly fall within the coverage domains of the special sciences. The structure of BFO, which indeed counts only 35 classes (http://ontology.buffalo.edu/bfo/BFO2.png), is based on a division of entities into two disjoint categories of continuant and occurrent, the former comprehending for example objects and spatial regions, the latter comprehending processes conceived as extended through (or as spanning) time. In this way, the BFO could constitute the top level classes of any ontology as all of them describe the same general reality, regardless of the scope,

Briefly, BFO organisation of reality is as follows:

1. **ENTITY -** anything that exists or has existed or will exist.
    - **CONTINUANT** - an entity that persists, endures, or continues to exist through time while maintaining its identity. Continuant can be divided in:
        - *independent continuant* - never depends on any other entity
        - *generically dependent continuant* - i.e. it depends on one or more other entities.
        - *specifically dependent continuant* - meaning it has a dependence, at a certain time, on some other specific independent entity.
    - **OCCURRENT** - is the philosophical development of a process that at any real point in time, like a snapshot, can be observed only for the part actually happening at that time. An example of occurrent is:
        - *process* - an occurrent that has a temporal actualisation and, at some point in time, it depends on some material entity.

## ONS: BROWSE AND LOOKUP ONS CONCEPTS

ONS is available online form various public repositories. One of the quickest and easiest ways to browse ONS classes is by using Bioportal (http://bioportal.bioontology.org/ontologies/ONS/?p=summary).

Upon opening, the Bioportal summary page for ONS is shown. This page reports some general information about ONS (i.e. the number of classes, the number of visits, and so on). The page also lists the version history (Bioportal retrieves changes from the ONS GitHub repository daily) and, more importantly, creates download links for the latest version of the ontology in various formats.

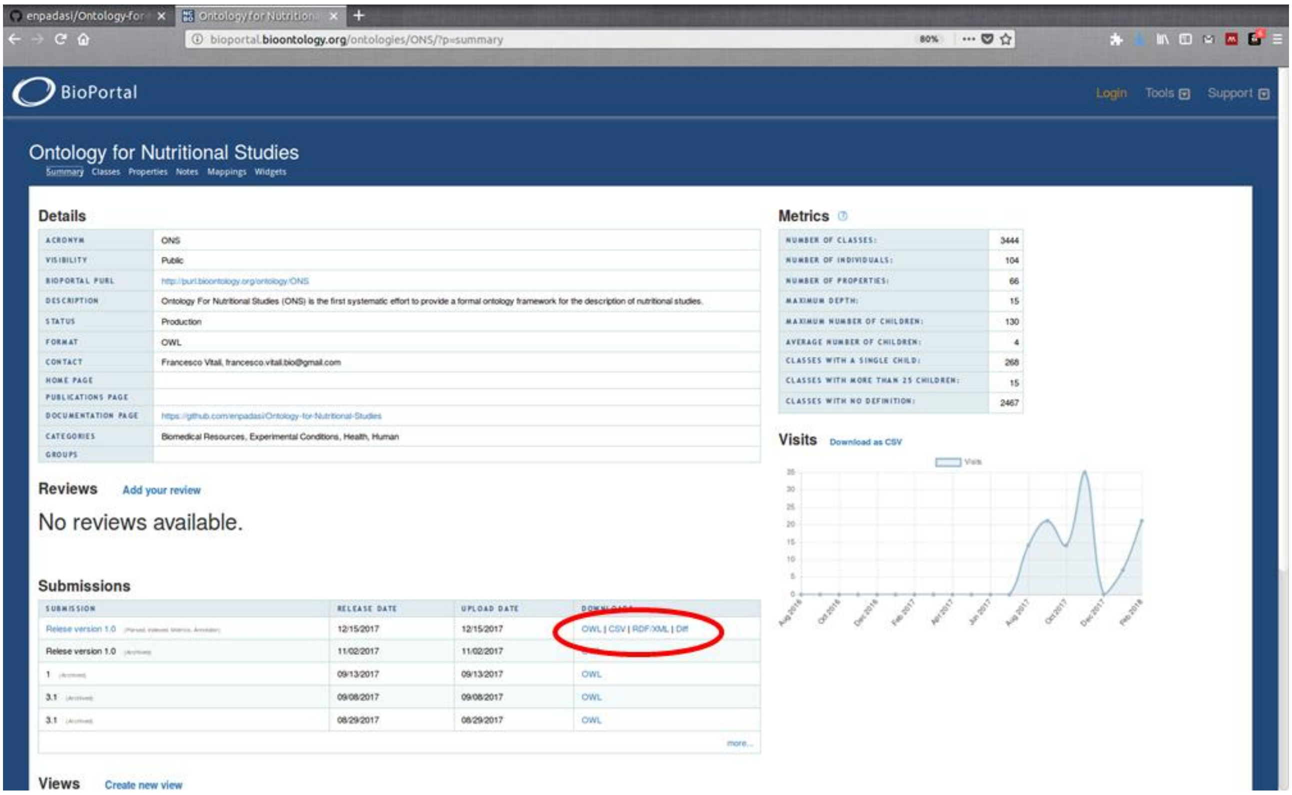


Figure 2: Screenshot of the Bioportal summary page for ONS. The red oval indicates the link to download ONS either in OWL or CSV format.

The user would be typically interested in either:

1. **CSV** format, to easily and quickly browse classes and definition in spreadsheet software. The resulting CSV file contains various columns mapping each class (column "Class ID") to various properties. In the "Class ID" column the URL of the class is reported, not particularly informative to humans. Typically the user would be interested in the columns:
    a. "Preferred label" which reports a human-readable label for the class
    b. "Definitions" reporting the textual definition of the class

Which are the most useful when searching ONS for a particular class. Other columns of the CSV file, reports the various properties of a class.

2. **OWL** format, to browse or make local changes using an ontology software, such as Protégé (see later for details)

ONS classes can be browsed by clicking on the "Classes" tab. Here, the left panel report the hierarchical organisation of ONS. The hierarchy of concepts can be expanded or reduced to show more general or more granular terms of the ontology. By clicking on a class in the hierarchy, the right panel shows information about the selected class (i.e. its definition, label, properties, etc.). The "Jump to" search tool allows searching for specific classes. Autocompletion functionality is available, so if the exact name of a class in ONS is not known, it can be found by typing few letters.

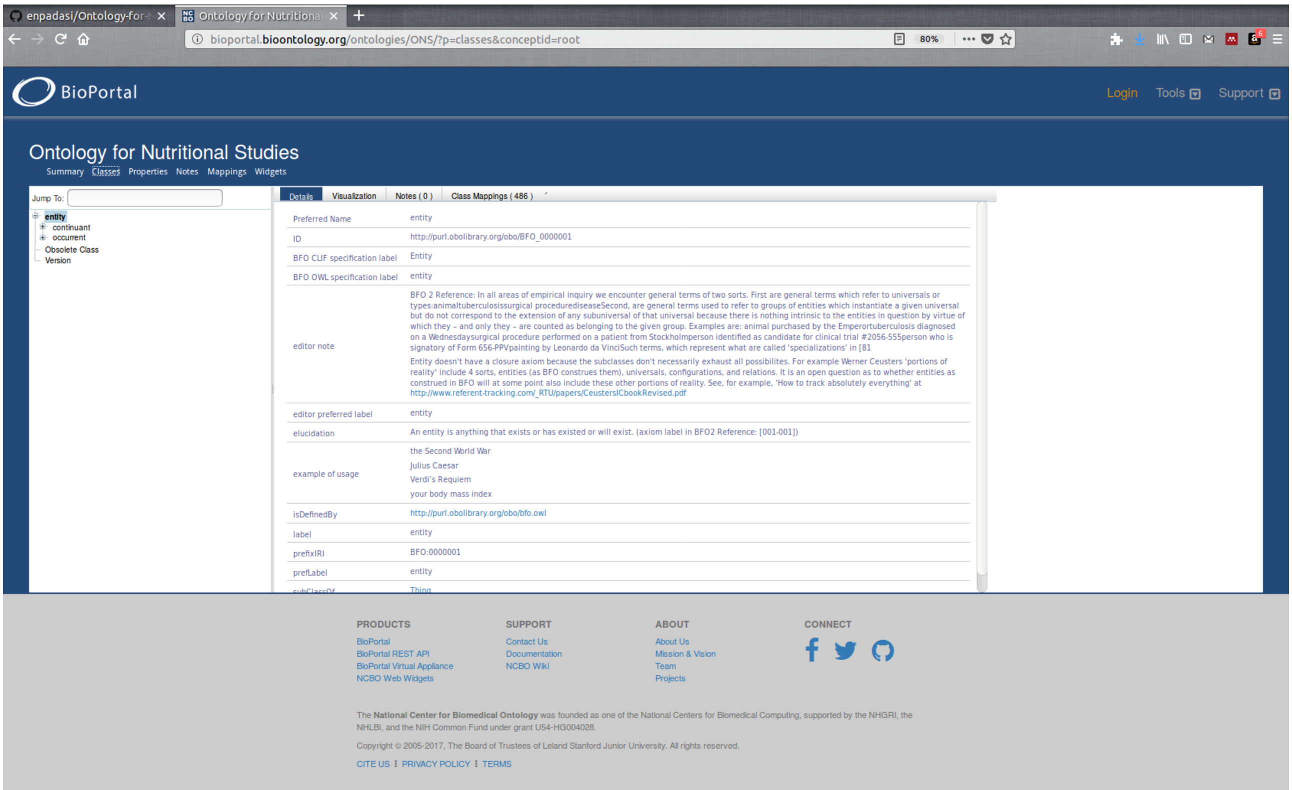


Figure 3: detail of the "Classes" tab in Bioportal for the "Entity" class.

## ONS: BROWSING AND MAKING CHANGES TO ONS LOCALLY

In this section, we will briefly explain how to browse and edit ONS using the software Protégé.

Protégé is one of the most widely used free and open-source ontology editor programs. It is supported by a wide and active community providing abundant sources for information on its usage (i.e. video tutorial, wiki pages, and proper courses). The first starting resource is the Protégé wiki pages (https://protegewiki.stanford.edu/wiki/Main_Page) but there are also some video tutorials on YouTube, visually illustrating the basic functionality and usage.

**Installation:** Protégé can be installed locally following information and instruction online (https://protege.stanford.edu/products.php#desktop-protégé). Protégé can also be used as its online version: Web Protégé (https://webprotege.stanford.edu/). In the latter case, the user would need to register to the site and upload the OWL file of ONS ontology (see later for download instruction)

**Download ONS:** To edit ONS locally using Protégé (but also online using WebProtégé), the user first need to download the ontology raw file. This can be done in various ways:

1. From the GitHub repository: the latest release version will always be located in enpadasi/Ontology-for-Nutritional-Studies/src/ontology/ (https://github.com/enpadasi/Ontology-for-Nutritional-Studies/tree/master/src/ontology). Only the .owl file is needed for importing ONS in Protégé, and this can be simply downloaded from here by right click -> save as

2. From the GitHub repository: alternatively, you can download the whole master branch of ONS repository from the main page, by clicking on “Clone or download” the “Download ZIP” in the menu that opens.

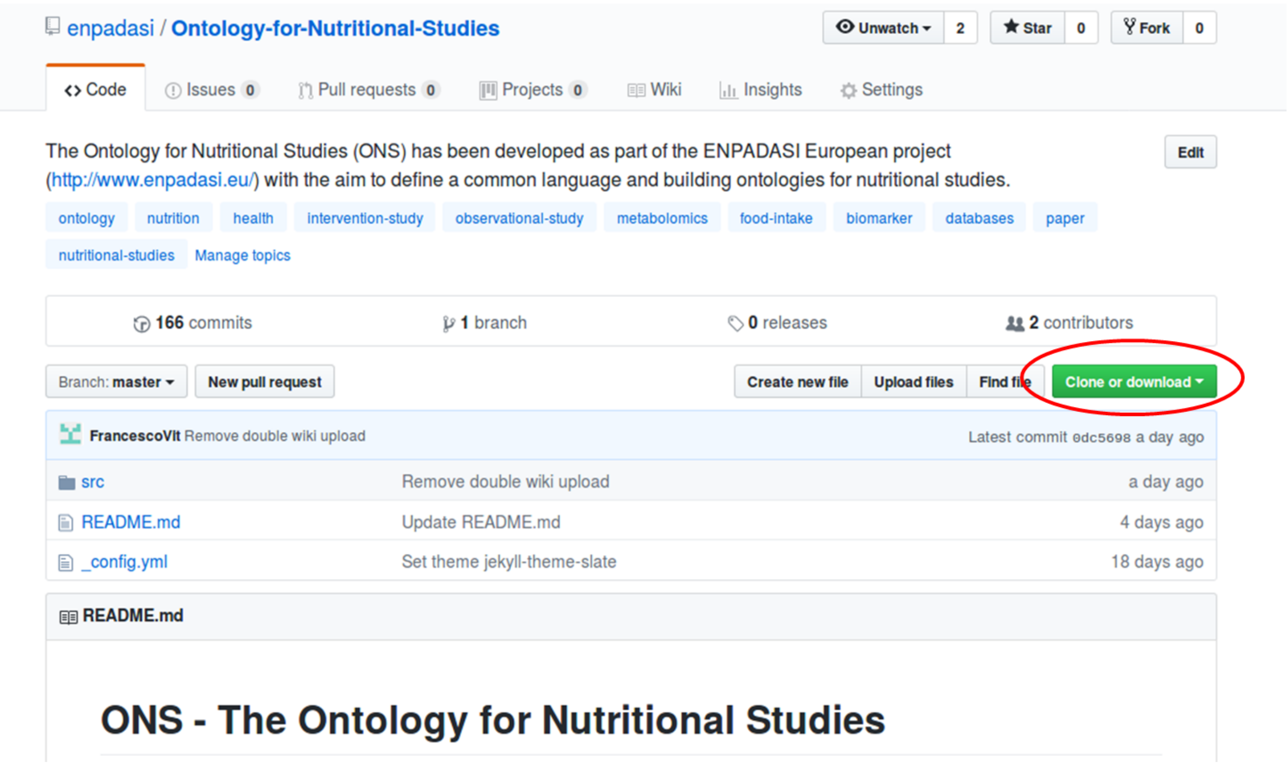


Figure 4: GitHub repository of ONS. The red oval indicates the link to download the entire repository.

3. From Bioportal: (see Figure 2) Upon opening, the Bioportal summary page for ONS is shown, reporting download link for the OWL format

The downloaded OWL file can be directly opened in Protégé (from the main window of the program select File -> Open... and browse to the location of the .owl file). Protégé will open the main ontology file, and automatically fetch from the GitHub repository the external ontology imports.

## ONS: CONTRIBUTE TO ONS

We encourage users to contribute to the ONS on every aspects of the ontology. The GitHub repository issue tracker is the preferred way of contribution to ONS. In fact, the GitHub issue tracker system makes all edit proposals traceable, and allows for open discussion among users and developers/maintainers on the proposed edit.

To contribute, the user firstly needs to possess a GitHub account (which can be created here https://github.com/) and log in. At this point, go to the main ONS repository page and click on the "Issues" tab.

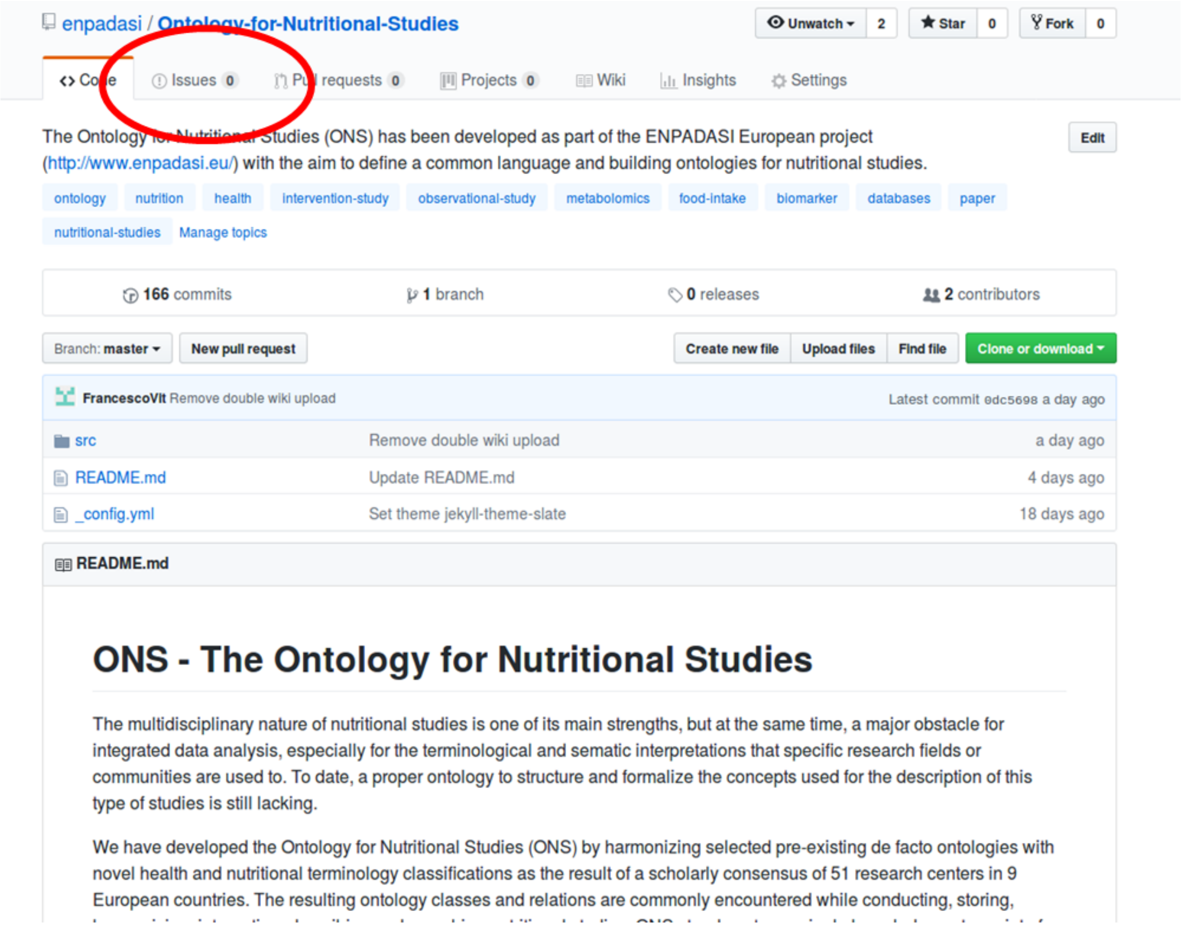


Figure 5: GitHub repository of ONS. The red oval indicates the Issues tab

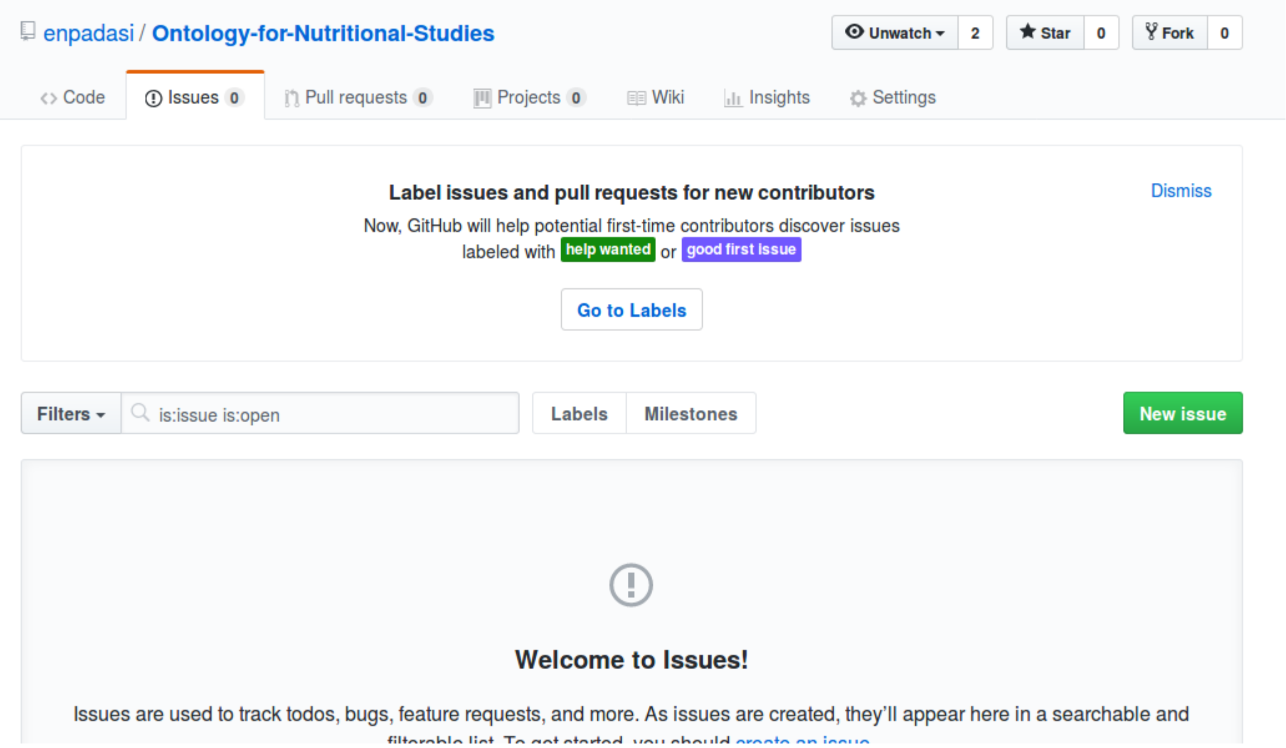


Figure 6: GitHub repository of ONS, Issue tab.

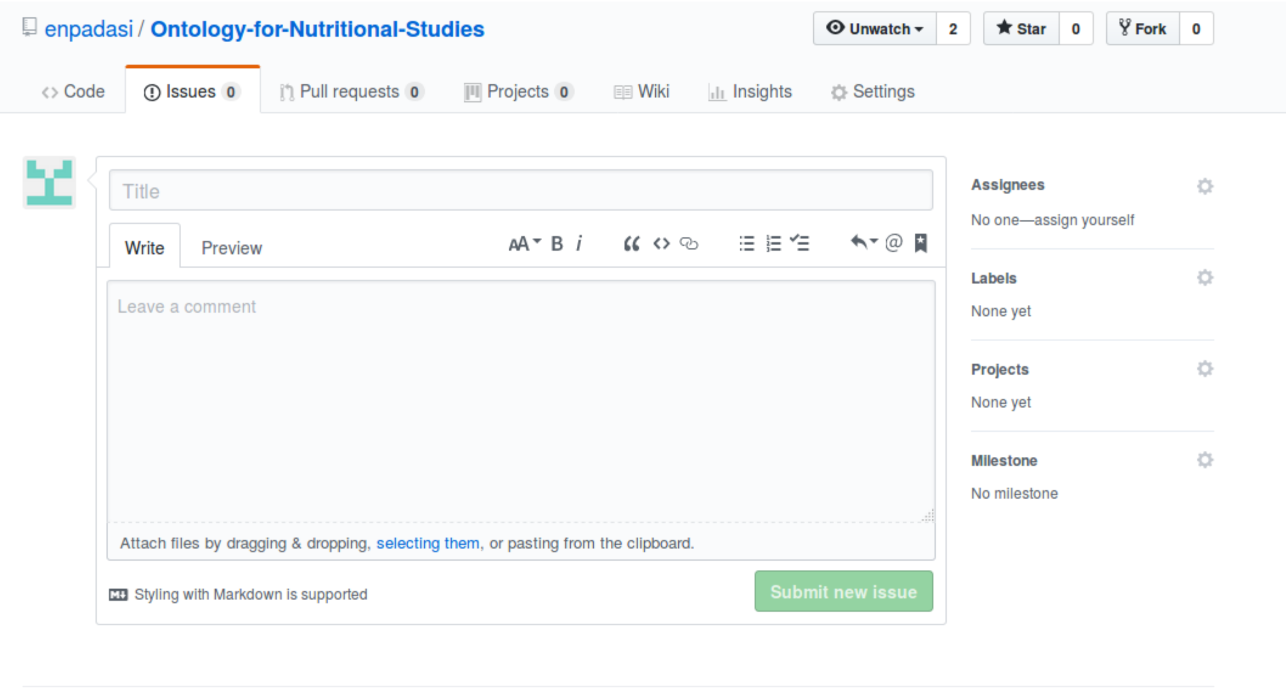


Figure 7: GitHub repository of ONS. Detail on the Issue submission page

In the Issue submission page (Figure 7), requirement/guidelines for an edit/improvement request are as follows:

1. Use tags in the title indicating the type of issue you are posting. Allowed tags (subjected to change) are:
    - [Existing term modification] to propose modification in an existing term/class. Every part of the term can be modified: definition, label, super-classes, relations
    - [New term request] to propose addition of a missing term/class.

Detail the requests following these templates, containing the minimum information necessary for a change:

**[Existing term modification]**

- Class label (i.e. Food)
- Class prefixed name (i.e. ONS_0000079)
- Item to edit (i.e. Textual definition, Relation with other classes, Annotation, other)
- Edit (indicate the proposed editing)

**[New term request]**

- Proposed label
- Proposed Super-class (to be identified among classes already inserted in ONS)
- Proposed Sub-class
- Proposed textual definition (i.e. the formal and technical English description that should be associated to the term).
- Proposed relations with other classes (propose in the form of triplet, preferably using properties already inserted in ONS)

After posting an issue, the developer will be notified and will process the request. Occasionally, both from the developers and from other users, a discussion about the proposed changes or additions can develop. This process eventually could lead to refine and improve the proposal, or other classes in ONS connected to it.
